# Editorial: Understanding the Operation of Visual Working Memory in Rich Complex Visual Context

**DOI:** 10.3389/fpsyg.2020.01996

**Published:** 2020-08-28

**Authors:** Hagit Magen, Marius V. Peelen, Tatiana Aloi Emmanouil, Zaifeng Gao

**Affiliations:** ^1^School of Occupational Therapy, The Hebrew University, Jerusalem, Israel; ^2^Donders Institute for Brain, Cognition and Behaviour, Radboud University, Nijmegen, Netherlands; ^3^Baruch College and the Graduate Center of the City University of New York, New York, NY, United States; ^4^Department of Psychology and Behavioral Sciences, Zhejiang University, Hangzhou, China

**Keywords:** visual working memory, gestalt, configuration, regularities, emotion, development, biological motion

As we move around in the world, we process a vast amount of visual information while we interact with objects or events, navigate our way in the environment, and process emotional and social information. Due to changes and interruptions in the visual input, for example during eye movements, task performance often relies on short-lived internal visual representations of the external world. Visual working memory (VWM) is the mechanism in charge of the formation and temporary maintenance of such representations (see Luck and Vogel, 2013; Ma et al., 2014, for reviews). Much work in the field of VWM has focused on identifying the basic units of VWM and the number of units that can be stored simultaneously, given its limited capacity (Cowan, 2001). Therefore, the majority of studies and theories in the field of VWM focus on the maintenance of basic, isolated, and often arbitrary visual stimuli, overlooking the involvement of VWM in the processing of highly rich and complex information, such as those frequently encountered in real life. The goal of the Research Topic was to broaden our knowledge regarding the maintenance of complex information in VWM. In the following sections, we provide a brief overview of the articles that appear in the collection. Among the issues addressed are: configural organization of VWM representations; metacognition for VWM performance; the role of emotions and of long-term memory (LTM) in VWM; and VWM for biological movements and real world objects.

Consistent with the structure present in natural environments, the basic representations of VWM are also structured, in a way that maintains not only individual item information but also configural properties of the entire display (Jiang et al., 2000; Brady et al., 2011). In this collection, Azer and Zhang adopted an individual differences approach to explore whether there is an association between configural processing in VWM and in perception. For each participant, the authors measured individual item and configural encoding in a VWM orientation task (Xie and Zhang, 2017) as well as holistic face processing using the composite face effect of the Le Grand face task (Le Grand et al., 2001). Configural encoding, but not individual item encoding, in VWM was correlated with holistic face processing. These findings support the hypothesis that perceiving configural properties in the environment leads to the encoding of these properties in VWM.

Most of the visual information we process is available simultaneously, affording the encoding of spatial configurations. However, information may also be accumulated over time, limiting the availability of spatial configural cues. Bharti et al. investigated the boundaries of configural processing in VWM by comparing the consequence of changing the locations of items between encoding and retrieval, when a set of binding stimuli was presented simultaneously or sequentially. In four experiments they consistently found that when a stimulus vanished as the next was presented, the WM advantage in the same-location condition over the randomized-location condition was dramatically reduced compared to the simultaneous presentation mode. These results suggest that WM encoding under the simultaneous presentation mode, but not under the sequential mode, relies on spatial configuration.

Magen and Emmanouil explored configural processing in self-initiated VWM, by examining the spatial structure of memory representations participants created for themselves, when space was task irrelevant. The results showed that participants constructed spatially structured representations for themselves and spatially unstructured representations for a hypothetical competitor in a memory contest. Nevertheless, in their explicit descriptions of the strategies they used, participants mentioned only non-spatial strategies. Thus, participants were guided by metacognitive knowledge on configural processing in VWM, knowledge that was implicit to some degree. The study shows that, mirroring the world, spatial structure is inherent to self-initiated VWM representations.

Metacognitive processing has gained little attention in VWM research. Sahar et al. explored how well individuals monitored their performance when maintaining real-world objects in VWM. Participants were tested independently on three dimensions: the objects' identity, location and temporal order. Monitoring was evaluated by comparing subjective confidence with objective performance. Similar biases in the subjective judgments of confidence were observed in all 3 dimensions, as reflected in overestimation of accuracy and underestimation of errors. Memory for real-world objects, which are represented in long-term memory (LTM), was enhanced relative to memory for meaningless images. Interestingly, monitoring of real-world objects was less biased.

Similarly investigating the influence of LTM on VWM, Zimmer and Fischer compared VWM for Chinese characters in Chinese and German participants. Across multiple experiments, they found that Chinese participants were better in detecting changes in the characters' shape but not in other aspects of these characters, such as their color or font type. These results provide novel evidence for an influence of LTM on VWM performance within the domain of word forms, contributing to a growing literature investigating the effects of familiarity and expertise on VWM performance (Jackson and Raymond, 2008; Curby et al., 2009; Kaiser et al., 2015; Xie and Zhang, 2017; Asp et al., 2019).

Only few studies investigated the developmental trajectory of VWM for complex materials. Guillory and Kaldy explored 12-month old infants' memory for real-world objects which were embedded in natural scenes. The authors examined whether, similar to adults, infants accumulate visual information in memory over time, and whether the formed memory representations are immune to interruptions. Infants' memory improved with longer exposures, showing accumulation of information over time. The formed representations were fragile as interference impaired memory performance. However, eye-tracking data demonstrated that some aspects of the scene survived the interference. The study adds to our understanding of the development of VWM in ecological contexts.

Three articles in the collection explored the interaction and role of VWM in emotion and social contexts. Costanzi et al. tested whether memory for spatial locations that were associated with emotional information was enhanced relative to locations that were associated with neutral stimuli. Moreover, they systematically manipulated the level of valence and arousal of the emotional stimuli in four experiments. The results demonstrated that emotions enhance spatial WM performance when neutral and emotional stimuli compete with one another for access into VWM, as well as shedded light on the interplay between arousal and valence in driving information processing into VWM.

Gambarota and Sessa provided a comprehensive review on the representation of faces in VWM, through its interaction with LTM and emotional and social cognitive mechanisms. They reviewed studies comparing VWM representations of faces and of other classes of stimuli, summarized the findings on representing static and changeable facial features in VWM, and finally examined research showing qualitative differences in VWM for face representations as a function of psychopathology and personality traits. This review enabled us to have a panorama about the representation of faces in VWM and its potential role in supporting socio-affective cognition.

Ye et al. further explored the function of retaining biological movements in VWM. Biological movements are one of the most complex stimuli in our daily life, and contain rich social information. The study revealed that VWM capacity for biological movements not only predicts core social ability (Gao et al., 2016; He et al., 2019), but also predicts canonical cognitive ability (e.g., fluid intelligence).

Taken together, the studies in this topic provide a more ecological view of VWM, seen as an adaptive system that evolved to reflect the structure of natural regularities, prioritize social, and emotional information which is necessary for survival, and integrates long-term knowledge of the environment. The articles highlight some of the important topics that need to be studied further and be incorporated into a comprehensive, more ecological model of VWM.

## Author Contributions

All authors listed have made a substantial, direct and intellectual contribution to the work and approved it for publication.

## Conflict of Interest

The authors declare that the research was conducted in the absence of any commercial or financial relationships that could be construed as a potential conflict of interest.

## References

[B1] Asp I. Störmer V. S. Brady T. F. (2019). Greater visual working memory capacity for visually-matched stimuli when they are recognized as meaningful. PsyArXiv [preprint]. 10.31234/osf.io/r6njf 34449847

[B2] Brady T. F. Konkle T. Alvarez G. A. (2011). A review of visual memory capacity: beyond individual items and toward structured representations. J. Vision 11, 1–34. 10.1167/11.5.4 21617025 PMC3405498

[B3] Cowan N. (2001). The magical number 4 in short-term memory: a reconsideration of mental storage capacity. Behav. Brain Sci. 24, 87–114. 10.1017/S0140525X01003922 11515286

[B4] Curby K. M. Glazek K. Gauthier I. (2009). A visual short-term memory advantage for objects of expertise. J. Exp. Psychol. Hum. Percept. Perform. 35, 94–107. 10.1037/0096-1523.35.1.94 19170473 PMC4159943

[B5] Gao Z. Ye T. Shen M. Perry A. (2016). Working memory capacity of biological movements predicts empathy traits. Psychon. Bull. Rev. 23, 468–475. 10.3758/s13423-015-0896-2 26174575

[B6] He J. Guo D. Zhai S. Shen M. Gao Z. (2019). Development of social working memory in preschoolers and its relation to theory of mind. Child Dev. 90, 1319–1332. 10.1111/cdev.13025 29292501

[B7] Jackson M. C. Raymond J. E. (2008). Familiarity enhances visual working memory for faces. J. Exp. Psychol. Hum. Percept. Perform. 34, 556–568. 10.1037/0096-1523.34.3.556 18505323 PMC4262787

[B8] Jiang Y. Olson I. R. Chun M. M. (2000). Organization of visual short-term memory. J. Exp.Psychol. Learn. Mem. Cogn. 26, 683–702. 10.1037/0278-7393.26.3.683 10855426

[B9] Kaiser D. Stein T. Peelen M. V. (2015). Real-world spatial regularities affect visual working memory for objects. Psychon. Bull. Rev. 22, 1784–1790. 10.3758/s13423-015-0833-4 25896215

[B10] Le Grand R. Mondloch C. J. Maurer D. Brent H. P. (2001). Early visual experience and face processing. Nature 410, 890–890. 10.1038/35073749 11309606

[B11] Luck S. J. Vogel E. K. (2013). Visual working memory capacity: from psychophysics and neurobiology to individual differences. Trends Cogn. Sci. 17, 391–400. 10.1016/j.tics.2013.06.006 23850263 PMC3729738

[B12] Ma W. J. Husain M. Bays P. M. (2014). Changing concepts of working memory. Nat. Neurosci. 17, 347–356. 10.1038/nn.3655 24569831 PMC4159388

[B13] Xie W. Zhang W. (2017). Discrete item-based and continuous configural representations in visual short-term memory. Vis. Cogn. 25, 21–33. 10.1080/13506285.2017.1339157

